# Dystonia and mitochondrial disease: the movement disorder connection revisited in 900 genetically diagnosed patients

**DOI:** 10.1007/s00415-024-12447-5

**Published:** 2024-05-22

**Authors:** Elisabetta Indelicato, Lea D. Schlieben, Sarah L. Stenton, Sylvia Boesch, Matej Skorvanek, Jan Necpal, Robert Jech, Juliane Winkelmann, Holger Prokisch, Michael Zech

**Affiliations:** 1 Institute of Human Genetics, Technical University of Munich, School of Medicine, Munich, Germany; 2 Institute of Neurogenomics, Helmholtz Munich, Deutsches Forschungszentrum Für Gesundheit Und Umwelt (GmbH), Ingolstädter Landstraße 1, 85764 Neuherberg, Germany; 3 Center for Rare Movement Disorders Innsbruck, Department of Neurology, Medical University Innsbruck, Innsbruck, Austria; 4 Division of Genetics and Genomics, Boston Children’s Hospital, Harvard Medical School, Boston, MA 02115 USA; 5 Program in Medical and Population Genetics, Broad Institute of MIT and Harvard, Cambridge, MA 02115 USA; 6 Department of Neurology, P. J. Safarik University, Kosice, Slovakia; 7 Department of Neurology, University Hospital of L. Pasteur, Kosice, Slovakia; 8 2nd Department of Neurology, Faculty of Medicine, Comenius University, Bratislava, Slovakia; 9 Department of Neurology, Zvolen Hospital, Zvolen, Slovakia; 10 Department of Neurology, Charles University in Prague, 1st Faculty of Medicine and General University Hospital in Prague, Kateřinská 30, 121 08 Prague, Czech Republic; 11 Munich Cluster for Systems Neurology (SyNergy), Munich, Germany; 12 Institute for Advanced Study, Technical University of Munich, Lichtenbergstrasse 2 a, 85748 Garching, Germany

## Introduction

Dystonia is characterized by excessive muscle contractions leading to abnormal posturing or movements, typically worsening with voluntary action and overflow muscle activation [1]. Dystonia can arise at any age and affect virtually any muscle [1]. The disease often co-occurs in combination with other movement disorders or additional neurological signs, resulting in a wide range of clinical outcomes [2]. Due to its puzzling pathophysiology, dystonia represents one of the core topics in movement disorders research [3]. High-throughput DNA sequencing has unveiled a strong genetic background for this condition [4], and has raised a number of novel questions, such as why dystonia-linked genes are implicated in multiple seemingly unrelated pathways [3]. These comprise but are not limited to a variety of metabolic processes, cellular ion handling, DNA binding and replication, signal transduction, neurotransmitter turnover, protein folding and intracellular trafficking [3]. This heterogeneous pathophysiology is consistent with a view of dystonia as a network disorder that is not associated with changes in a single neural pathway, but may result from disturbances in multiple regions of the nervous system [3]. In contrast to Parkinson’s disease [5], a pathophysiological link between monogenic dystonias and the far more frequent ‘idiopathic’ adult-onset isolated dystonias is missing.

Mitochondrial dysfunction has been identified as a relevant pathophysiological factor in dystonia [6] and mitochondrial DNA (mtDNA) defects have been linked to pathognomonic dystonia syndromes in an association dubbed “the movement disorder connection” [6]. This term was coined following the observation that defects in several mitochondrial pathways can all lead to the clinical outcome of dystonia, raising the hypothesis that mitochondrial defects in general may be involved in the pathogenesis of movement disorders [6]. Beyond the 37 proteins and RNAs encoded by the mtDNA, more than 1500 nuclear encoded proteins are involved in mitochondrial structure and function [7]. It is nowadays established that defects in all these factors may result in mitochondrial dysfunction, leading to a new concept of mitochondrial disease genes [7]. Movement disorders, including dystonia, are a frequent manifestation of nuclear-encoded mitochondrial defects as shown by numerous case reports and corroborated by a few smaller case series [8–11]. However, the implication of nuclear mitochondrial disease genes in dystonia has not yet been assessed systematically at a cohort-wide level [3]. This unresolved issue along with scarce experimental evidence hinders firm conclusions to be drawn on the contribution of mitochondrial dysfunction to dystonia pathophysiology. Understanding the role of mitochondrial impairment in larger numbers of dystonia-affected individuals may also have relevance for the more common isolated dystonias presenting to the adult neurology clinic. Early studies hint toward mitochondrial dysfunction in ‘idiopathic’ dystonia [12, 13], however, these have not been followed by in-depth replication. Recent reports describing isolated dystonia phenotypes in the setting of complex V defects prompted us to reexamine the contribution of mitochondrial defects in the wider spectrum of dystonic diseases [14] and vice versa the frequency of dystonia in pediatric mitochondrial disease cases [15].

Herein, we explored the contribution of nuclear-encoded mitochondrial disease gene defects to dystonia from a cohort-level perspective. The study analyzed two large collections of individuals who underwent whole exome sequencing (WES) for the indications of (i) dystonia and (ii) clinically suspected mitochondrial disease. The aim of this study was to define (1) the frequency of mitochondrial disease gene defects in cases ascertained for dystonia, (2) the prevalence of dystonia as a clinical feature in patients with genetically diagnosed mitochondrial disease, and (3) whether specific molecular pathways related to mitochondrial dysfunction are more often perturbed in each of these settings.

## Methods

### Study population

Within a multicenter study on the monogenic background of dystonia a total of 1,100 index cases with dystonic disorders were assessed for mitochondrial disease gene defects using WES (patients previously published in [2]). Both adults (≥ 21 years, 67%) and children/adolescents (< 21 years, 33%) were included [2]. A molecular diagnosis was identified in 220 cases within the dystonia cohort [2]. Patients with clinically suspected mitochondrial disease were extracted from the GENOMIT consortium, as previously described [16]. Within GENOMIT, 2023 pediatric patients with at least a “possible” diagnosis of mitochondrial disease according to the Nijmegen Mitochondrial Disease Criteria [17] were recruited and genetically tested using WES. Details on the study protocol are provided in Stenton et al. [15]. Within the cohort, 1,109 patients (55%) received a molecular diagnosis, of whom 683 cases were solved by the detection of variants in nuclear-encoded mitochondrial genes. The here-evaluated patients have been reported in our previous cohort studies on genetic dystonias and mitochondrial disorders [2, 15, 16].

### Definition of nuclear mitochondrial disease genes

Mitochondrial disease genes were grouped into seven previously published functional subcategories [7, 15]: (1) oxidative phosphorylation (OXPHOS) subunits, assembly factors, and electron carriers, (2) mtDNA replication and expression, (3) mitochondrial dynamics, homeostasis, and quality control, (4) metabolism of substrates, (5) metabolism of cofactors, (6) metabolism of toxic compounds and (7) others/unknown function. For this study, we focused solely on nuclear-encoded mitochondrial disease genes due to the applied testing design of the study cohorts: all patients underwent WES with targeted analysis of the nuclear genome. The mitochondrial genome was not analyzed in the dystonia samples (mtDNA calling pipeline not available for all cases). We therefore gave priority to analysis of nuclear gene defects in this study.

## Results

Among 220 genetically resolved dystonia exomes [2], 29 patients had disease-causing variants in nuclear-encoded mitochondrial disease genes (13%), spanning 19 different genes (Table 1). The most frequent mitochondrial disease gene hits were *PANK2* (6/29 cases, 21%), *WARS2*, and *COQ8A* (each with 3/29 cases, 10%). The majority of diagnosed gene etiologies (15/19 genes) were reported in single cases only. The largest gene cluster was related to “mitochondrial dynamics, homeostasis and quality control” (8/19 genes, including: *AFG3L2, DNM1L, OPA1, PINK1, PRKN, SERAC1, SPG7, VPS13D*), followed by genes directly related to OXPHOS composition, cofactors, or assembly. The majority of genetic defects were inherited in an autosomal recessive manner (79%), while in six out of 29 patients (21%) the mode of inheritance was autosomal dominant. Five patients (one each with variants in *COQ8A, OPA1, PINK1, SPG7, WFS1)* had adult-onset dystonia, while the other 24 patients had an onset of dystonia before the age of 21. With regard to clinical presentation, most of the patients (20/29 cases, 69%) fell into the category of “complex” dystonia, presenting with additional non-movement disorder features such as intellectual disability or epilepsy [2]. Seven patients displayed dystonia combined with another movement disorder (ataxia, parkinsonism, spasticity) [2]. Only one patient carrying compound heterozygous *COQ8A* variants presented with isolated dystonia. Of the 19 mutated genes, five are not formally linked to a dystonia phenotype in OMIM (*SPG7, DNM1L, NARS2, OPA1, WFS1*). There are however cumulative reports in the literature supporting an association with dystonia for at least 4 of these genes [18–21].

**Table 1:** Nuclear-encoded mitochondrial disease gene diagnoses identified in solved dystonia cases

Functional Category	Gene	Mode of inheritance	Dystonia category	n. of cases
OXPHOS subunits, assembly factors, and electron carriers	*ATP5F1A*	AD	*Complex* (ID, ataxia, spasticity)	1
	*ATP5MC3*	AD	*Combined* (spasticity)	1
	*COQ8A*	AR	*isolated/combined* (ataxia)	3
	*SCO2*	AR	*Complex* (ID)	1
Mitochondrial DNA replication and expression	*NARS2*	AR	*Complex* (epilepsy)	1
	*WARS2*	AR	*Complex* (ID, parkinsonism)	3
Mitochondrial dynamics, homeostasis, and quality control	*AFG3L2*	AD	*Complex* (ID, ataxia)	1
	*DNM1L*	AD	*Complex* (ID, epilepsy)	1
	*OPA1*	AD	*Complex* (optic atrophy)	1
	*PINK1*	AR	*Combined* (parkinsonism)	1
	*PRKN*	AR	*Combined* (parkinsonism)	1
	*SERAC1*	AR	*Complex* (ID)	1
	*SPG7*	AR	*Combined* (ataxia)	1
	*VPS13D*	AR	*Complex* (ID, ataxia)	1
Metabolism of substrates	***FA2H***	AR	*Complex* (ID, spasticity)	1
Metabolism of cofactors	*MECR*	AR	*Complex* (optic atrophy)	1
	***PANK2***	AR	*Complex* (ataxia, spasticity, cognitive decline)	6
	***PLA2G6***	AR	*Combined/complex* (ataxia, ID)	2
	*WFS1*	AD	*Complex* (optic atrophy, deafness, migraine)	1

Genes associated with NBIAs are shown in bold. While some genes may be associated with both autosomal recessive (AR) and autosomal dominant inheritance (AD), we herein reported only the mode of inheritance specific to the variants detected in the present study. ID: intellectual disability

Out of 683 solved exomes of pediatric patients with nuclear-encoded mitochondrial gene-related disease, 83 cases presented clinically with dystonia (12%). The molecular diagnoses spanned 45 different mitochondrial disease genes, of which more than half (n = 27) were detected in single cases only (Table 2). The most frequent genetic etiologies were *ECHS1*-related disorder (16/83 cases, 19%), as well as *NDUFAF6*- (4 cases), *COQ4*-, *PANK2*- and *SERAC1*-related diseases (each with 3 cases). Considering the different functional pathways, the largest gene category in this cohort was the OXPHOS subunits or assembly factors (n = 12), followed by genes involved in the metabolism of cofactors (n = 10). All patients with mitochondrial gene-related disease displayed an autosomal recessive mode of inheritance, with the exception of one X-linked case. Regarding the clinical presentation, all 83 patients fell in the category of “complex dystonia”.

**Table 2:** Nuclear genes implicated in genetically solved mitochondrial disease cases presenting with dystonia

Functional category	Gene	Mode of inheritance	Neurological features other than dystonia	Extraneurological features	n. of cases
OXPHOS subunits, assembly factors, and electron carriers	*ATP5F1E*	AR	ID, ataxia, neuropathy		2
	*ATP5PO*	AR	DD, hypotonia, epilepsy, microcephaly		1
	*BCS1L*	AR	DD, hypotonia, ID, epilepsy, myoclonus, spasticity	Hypoglycemia, gastrointestinal dysmotility, lactic acidosis,	1
	*COQ4*	AR	DD, hypotonia, ID, epilepsy, microcephaly	Facial dysmorphism	3
	*COX10*	AR	DD, hypotonia, ataxia	Failure to thrive, lactic acidosis	1
	*DNAJC30*	AR	DD, ataxia		1
	*FOXRED1*	AR	DD, epilepsy, myoclonus, visual impairment	Lactic acidosis	1
	*LYRM7*	AR	DD/ DR, ID, hypotonia, microcephaly, deafness, visual impairment, tremor	Failure to thrive, hypoglycemia, facial dysmorphism, lactic acidosis, exercise intolerance	1
	*NDUFA12*	AR	DD/DR, ID, hypotonia, spasticity, visual impairment, microcephaly, tremor, neuropathy	Lactic acidosis, scoliosis	2
	*NDUFA4*	AR	DD/DR, ataxia	Failure to thrive, lactic acidosis, hypertrophic cardiomyopathy, myopathy, exercise intolerance	1
	*NDUFAF5*	AR	DD/DR, ataxia	Lactic acidosis	2
	*NDUFAF6*	AR	DD/DR, spasticity, ataxia, visual impairment	Gastrointestinal dysmotility, lactic acidosis, scoliosis	4
Mitochondrial DNA replication and expression	*GFM1*	AR	DD, epilepsy, spasticity, neuropathy, visual impairment	Muscular dystrophy, lactic acidosis	1
	*GFM2*	AR	DD/DR, hypotonia, ID, epilepsy, microcephaly, spasticity, neuropathy	Failure to thrive, gastrointestinal dysmotility, lactic acidosis, muscular dystrophy, exercise intolerance	1
	*MRPS34*	AR	DD, ID, microcephaly		1
	*MTFMT*	AR	DD, ID, hypotonia, ataxia, attention deficit-hyperactivity disorder	Failure to thrive, lactic acidosis, anemia, strabismus, hypertrophic cardiomyopathy	2
	*PNPT1*	AR	DR, ID		2
	*SARS2*	AR	DD/DR, hypotonia, spasticity	Lactic acidosis, cataract	1
	*WARS2*	AR	DD, ID, athetosis, hemiballismus,		1
Mitochondrial dynamics, homeostasis, and quality control	*CLPB*	AR	hypotonia, epilepsy	Neutropenia, anemia, apneas	1
	*SERAC1*	AR	DD, hypotonia, ID, spasticity, microcephaly, deafness, epilepsy, ataxia, optic atrophy	Failure to thrive, facial dysmorphism, lactic acidosis, hypoglycemia, kidney dysfunction, liver failure	3
	*SPG7*	AR	DD, ID, ataxia		1
	*TIMM50*	AR	DD, epilepsy, spasticity, optic atrophy	Failure to thrive, neutropenia, cardiomyopathy, lactic acidosis, scoliosis	1
	*YME1L1*	AR	DR, ataxia	Lactic acidosis, cardiomyopathy, exercise intolerance	1
Metabolism of substrates	*ALDH18A1*	AR	DD, hypotonia, ID, epilepsy, microcephaly, spasticity	Failure to thrive, cataract	1
	***FA2H***	AR	DD, ID, ataxia		2
	*MDH2*	AR	DD, epilepsy, hypotonia	Failure to thrive, lactic acidosis	1
	*PDHA1*	X-linked	DD, spasticity, visual impairment	Lactic acidosis	2
Metabolism of cofactors	***COASY***	AR	DD, ID, ataxia, spasticity		1
	*FDXR*	AR	hypotonia, ID, epilepsy, ataxia, spasticity, optic atrophy, visual impairment, deafness, ophthalmoplegia, neuropathy	Myopathy, diabetes mellitus	1
	*ISCA2*	AR	DD/DR, epilepsy, spasticity	Anemia, lactic acidosis	1
	*LIPT2*	AR	DD/DR, hypotonia, ID, microcephaly, epilepsy, spasticity	Lactic acidosis	2
	*MECR*	AR	DD		1
	***PANK2***	AR	DD, ID, ataxia, spasticity, tremor		3
	*SEPSECS*	AR	DD, ID		1
	*SLC19A3*	AR	DD, ataxia		2
	*SLC25A42*	AR	DD, hypotonia, ID, macrocephaly, choreoathetosis	Rhabdomyolysis, failure to thrive, gastrointestinal dysmotility, facial dysmorphism, lactic acidosis	2
	*TPK1*	AR	DD	Feeding difficulties, muscular dystrophy	1
Metabolism of toxic compounds	*ECHS1*	AR	DD/DR, ID, hypotonia, microcephaly, epilepsy, spasticity, ataxia, deafness, neuropathy, optic atrophy, stroke-like episodes,	Failure to thrive, gastrointestinal dysmotility, lactic acidosis, diabetes insipidus, abnormality of temperature regulation, hypertrophic cardiomyopathy, kidney dysfunction, exercise intolerance, hepatomegaly, apneas	16
	*ETHE1*	AR	DD/DR, myoclonus, ataxia, spasticity	Lactic acidosis	1
	*HIBCH*	AR	DD/DR	Failure to thrive	1
Others/Unknown function	***C19orf12***	AR	DD, ID, ataxia		2
	*FBXL4*	AR	DD, ID, hypotonia, spasticity		1
	***PLA2G6***	AR	DD, ID, ataxia		3
	*SPATA5*	AR	DD, epilepsy, microcephaly, myoclonus, spasticity, deafness, visual impairment	Failure to thrive, lactic acidosis	2

Genes associated with NBIAs are shown in bold. While some genes may be associated with both autosomal recessive (AR) and autosomal dominant inheritance (AD), we herein reported only the mode of inheritance specific to the variants detected in the present study. DD: developmental delay; DR: developmental regression; ID: intellectual disability

The only gene category showing a tendency towards more frequent implication in the dystonia cohort, as compared to the mitochondrial disease cohort, was that related to “mitochondrial dynamics, homeostasis, and quality control” (category 3). Six gene etiologies (*FA2H, PANK2, PLA2G6, SERAC1, SPG7*, and *WARS2*) were identified both in the dystonia and in the mitochondrial disease cohort. Three of these genes (*FA2H, PANK2, PLA2G6)* are associated with a group of disorders known as NBIAs (neurodegeneration with brain iron accumulation).

## Discussion

Herein, we systematically re-evaluated two large WES cohorts to delineate the contribution of nuclear-encoded mitochondrial disease genes to the clinical expression of dystonia. We found that variants in nuclear-encoded mitochondrial disease genes accounted for 13% of diagnoses in 220 WES-solved dystonia cases, whereas 12% of 683 pediatric cases with monogenic mitochondrial disease due to variants in nuclear-encoded mitochondrial disease genes had dystonia. Overall, variants in 57 nuclear-encoded mitochondrial disease genes were involved in a dystonia phenotype in the two explored large patient collections.

Previous case series [8–11] report a variable frequency of dystonia in mitochondrial disease cohorts, ranging from 0.8% to 10%. Differences may be explained by heterogeneous recruitment criteria for genetic testing and variable representation of pediatric versus adult cases. Furthermore, the diagnosis of dystonia within early-onset severe multisystemic phenotypes (such as most mitochondriopathies) is challenging, likely resulting in unreported individuals [22]. By analyzing a large cohort referred for WES with the primary indication of dystonia, we also tackled the research question from the opposite perspective. Our estimation based on this cohort might, therefore, better reflect the “real-world” frequency of nuclear-encoded mitochondrial etiologies in monogenic dystonias. Nevertheless, our findings may still underestimate the overall burden of mitochondrial defects in the pathogenesis of dystonia, as we specifically addressed nuclear gene defects in our cohort. In the present study and in previous cohorts [8–11], dystonia mostly occurred within complex phenotypes, as expected considering the pleiotropic effect of mitochondrial dysfunction. Unbiased sequencing increasingly allows detection of mitochondrial defects in isolated dystonia cases [3]. In the dystonia cohort studied here, one index case with isolated dystonia harbored compound heterozygous disease-causing *COQ*8 variants. Indeed, isolated writer’s cramp is a recurrent early feature in the multisystemic *COQ8*-related disease spectrum [23]. Recently, heterozygous variants in complex V-encoding genes, including *ATP5F1B*, have also been linked to isolated dystonia [24].

The greatest diagnostic overlap between the dystonia cohort and the mitochondrial disease cohort was in genes classically associated with NBIAs, a group of disorders sharing the hallmark of pathological iron deposition in the basal ganglia [25]. Interestingly, NBIA is usually caused by defects in proteins with no direct role in iron metabolism, but often with an established function in mitochondrial pathways. Patients with NBIA are therefore often included in mitochondrial disease cohorts [25]. The clinical presentation of NBIAs is very heterogeneous, spanning from early-onset severe neurodevelopmental phenotypes to juvenile-onset dystonia and/or parkinsonism. The characteristic basal ganglia hypointensities in iron-sensitive MRI sequences may be subtle or not present at all at the time of genetic diagnosis [25]. Thus, unbiased WES can decisively contribute to the identification of these conditions in a “genetics first” approach. This may explain the relative high frequency of these rare and ultra-rare diagnoses in our study and the different landscape of identified variants as compared to earlier case series studying genetic mitochondrial-disease contributions [8–11] In this light, it might be anticipated that newer technologies, e.g. genome-wide long-read sequencing with analysis of non-coding gene regions, would again reshape the genetic understanding of such disorders and improve the yield of genetic testing [26].

The awareness of an underlying mitochondrial defect in patients with dystonia has a number of implications in daily clinical practice, such as the avoidance of specific drugs and dedicated perioperative measures. Moreover, sparse literature suggests a relevant role of mitochondrial disease genes in children presenting with the life-threatening complication of status dystonicus [27, 28].

Dissecting the contribution of mitochondrial dysfunction to monogenic dystonias may guide investigations on the pathophysiology of more common forms of adult-onset isolated dystonia [14, 29]. On the one hand, subtle alterations in mitochondrial function may produce an endophenotype which determines a susceptibility in key networks [29]. On the other hand, declining mitochondrial quality due to impaired biogenesis and mitophagy, as seen with aging [30], may act as “second hit” in a susceptible genetic background [14]. Notably, variants in genes related to mitochondrial dynamics, homeostasis, and quality control (category 3 in our study) are a recurrent cause of monogenic movement disorders with adult onset [18, 31, 32]. Across different disorders, defects in mitochondrial pathways share common consequences at the cellular level consisting of oxidative stress, iron dysmetabolism and inflammation [30, 33, 34]. Targeting these pleiotropic downstream effects represents an attractive approach with potential application in different diseases. The pharmacological modulation of the transcription factor *NRF2* is an example of such strategy [35]. In response to various stimuli, *NRF2* binds specific “antioxidant response elements” in the promoter region of at least 250 target genes and activates a plethora of cellular processes aiming at preserving the cellular redox homeostasis and mitochondrial function. A *NRF2* activator has been recently approved for the treatment of the nuclear gene-related mitochondrial disease Friedreich’s ataxia [35]. The modulation of *NRF2* has been repurposed also for the treatment of Parkinson´s disease and multiple sclerosis [33]. For dystonia, in-depth studies on the mechanistic events connecting mitochondrial dysfunction with this clinical phenotype are urgently needed to prioritize therapeutic targets for further investigation.

## References

[1] Albanese A, Bhatia K, Bressman SB (2013). Phenomenology and classification of dystonia: a consensus update. Mov Disord.

[2] Dzinovic I, Boesch S, Škorvánek M (2022). Genetic overlap between dystonia and other neurologic disorders: a study of 1,100 exomes. Park Relat Disord.

[3] Jinnah HA, Sun YV (2019). Dystonia genes and their biological pathways. Neurobiol Dis.

[4] Zech M, Jech R, Boesch S (2020). Monogenic variants in dystonia: an exome-wide sequencing study. Lancet Neurol.

[5] Blauwendraat C, Nalls MA, Singleton AB (2020). The genetic architecture of Parkinson’s disease. Lancet Neurol.

[6] Wallace DC, Murdock DG (1999). Mitochondria and dystonia: the movement disorder connection?. Proc Natl Acad Sci U S A.

[7] Schlieben LD, Prokisch H (2020). The dimensions of primary mitochondrial disorders. Front Cell Dev Biol.

[8] Schreglmann SR, Riederer F, Galovic M (2018). Movement disorders in genetically confirmed mitochondrial disease and the putative role of the cerebellum. Mov Disord.

[9] Ticci C, Orsucci D, Ardissone A (2021). Movement disorders in children with a mitochondrial disease: a cross-sectional survey from the nationwide Italian collaborative network of mitochondrial diseases. J Clin Med.

[10] Montano V, Orsucci D, Carelli V (2022). Adult-onset mitochondrial movement disorders: a national picture from the Italian Network. J Neurol.

[11] Martikainen MH, Ng YS, Gorman GS (2016). Clinical, genetic, and radiological features of extrapyramidal movement disorders in mitochondrial disease. JAMA Neurol.

[12] Schapira AHV, Warner T, Gash MT (1997). Complex I function in familial and sporadic dystonia. Ann Neurol.

[13] Benecke R, Strümper P, Weiss H (1992). Electron transfer complex I defect in idiopathic dystonia. Ann Neurol.

[14] Indelicato E, Boesch S, Mencacci NE (2024). Dystonia in ATP synthase defects: reconnecting mitochondria and dopamine. Mov Disord.

[15] Stenton SL, Shimura M, Piekutowska-Abramczuk D, et al (2021) Diagnosing pediatric mitochondrial disease: lessons from 2,000 exomes. medRxiv 2021.06.21.21259171. 10.1101/2021.06.21.21259171

[16] Zech M, Kopajtich R, Steinbrücker K (2022). Variants in mitochondrial ATP synthase cause variable neurologic phenotypes. Ann Neurol.

[17] Morava E, Van Den Heuvel L, Hol F (2006). Mitochondrial disease criteria: diagnostic applications in children. Neurology.

[18] Van Gassen KLI, Van Der Heijden CDCC, De Bot ST (2012). Genotype-phenotype correlations in spastic paraplegia type 7: a study in a large Dutch cohort. Brain.

[19] Ortega-Suero G, Fernández-Matarrubia M, López-Valdés E, Arpa J (2019). A novel missense OPA1 mutation in a patient with dominant optic atrophy and cervical dystonia. Mov Disord Clin Pract.

[20] Keller N, Paketci C, Edem P (2021). De novo DNM1L variant presenting with severe muscular atrophy, dystonia and sensory neuropathy. Eur J Med Genet.

[21] Finsterer J, Mehri S (2023). Progressive mitochondrial encephalopathy due to the novel compound heterozygous variants c.182C>T and c.446A>AG in NARS2: a case report. Cureus.

[22] Koens LH, Klamer MR, Sival DA (2023). A screening tool to quickly identify movement disorders in patients with inborn errors of metabolism. Mov Disord.

[23] Amprosi M, Zech M, Steiger R (2021). Familial writer’s cramp: a clinical clue for inherited coenzyme Q10 deficiency. Neurogenetics.

[24] Nasca A, Mencacci NE, Invernizzi F (2023). Variants in ATP5F1B are associated with dominantly inherited dystonia. Brain.

[25] Hayflick SJ, Kurian MA, Hogarth P (2018). Neurodegeneration with brain iron accumulation. Handb Clin Neurol.

[26] Sturchio A, Marsili L, Mahajan A (2020). How have advances in genetic technology modified movement disorder nosology?. Eur J Neurol.

[27] Lumsden DE, Cif L, Capuano A, Allen NM (2023). The changing face of reported status dystonicus—a systematic review. Park Relat Disord.

[28] Saini AG, Hassan I, Sharma K (2022). Status dystonicus in children: a cross-sectional study and review of literature. J Child Neurol.

[29] Rauschenberger L, Knorr S, Pisani A (2021). Second hit hypothesis in dystonia: dysfunctional cross talk between neuroplasticity and environment?. Neurobiol Dis.

[30] Lima T, Li Y, Mottis A, Auwerx J (2022). Pleiotropic effects of mitochondria in aging. Nat Aging.

[31] Jia F, Fellner A, Kumar KR (2022). Monogenic Parkinson’s disease: genotype, phenotype, pathophysiology, and genetic testing. Genes (Basel).

[32] Di Bella D, Lazzaro F, Brusco A (2010). Mutations in the mitochondrial protease gene AFG3L2 cause dominant hereditary ataxia SCA28. Nat Genet.

[33] Dodson M, De La Vega MR, Cholanians AB (2019). Modulating NRF2 in disease: timing is everything. Annu Rev Pharmacol Toxicol.

[34] Pilotto F, Chellapandi DM, Puccio H (2024). Omaveloxolone: a groundbreaking milestone as the first FDA-approved drug for Friedreich ataxia. Trends Mol Med.

[35] Boesch S, Indelicato E (2024). Approval of omaveloxolone for Friedreich ataxia. Nat Rev Neurol.

